# 125 Pain Management in Adult and Pediatric Burns: A Global Survey of Burn Care Providers

**DOI:** 10.1093/jbcr/irae036.124

**Published:** 2024-04-17

**Authors:** Cameron Gibson, Morgan Dewey, Kori Baker, Scott W Mueller, Blaire Balstad

**Affiliations:** University of Colorado, Aurora, CO; UCHealth and University of Colorado School of Pharmacy, Aurora, CO; University of Colorado Anschtuz, Denver, Colorado; University of Colorado, Aurora, CO; UCHealth and University of Colorado School of Pharmacy, Aurora, CO; University of Colorado Anschtuz, Denver, Colorado; University of Colorado, Aurora, CO; UCHealth and University of Colorado School of Pharmacy, Aurora, CO; University of Colorado Anschtuz, Denver, Colorado; University of Colorado, Aurora, CO; UCHealth and University of Colorado School of Pharmacy, Aurora, CO; University of Colorado Anschtuz, Denver, Colorado; University of Colorado, Aurora, CO; UCHealth and University of Colorado School of Pharmacy, Aurora, CO; University of Colorado Anschtuz, Denver, Colorado

## Abstract

**Introduction:**

Pain management for burn injuries is an essential component of compassionate burn care. Practices may vary by region or socioeconomic status. This study aimed to assess current pain management practices in burn patients globally.

**Methods:**

An online survey regarding the availability and use of drugs for burn pain management was sent to members of the International Society for Burn Injuries (ISBI) via email and shared in WhatsApp groups comprised of burn professionals. The primary outcome was to provide a cumulative data representation to increase knowledge regarding the state of global burn pain management. Data was analyzed for descriptive statistics by region and socioeconomic status.

**Results:**

113 out of 197 surveys were completed (57%) from 44 different countries. 80% of respondents were MD’s, 15% RNs, and 5% other. By region, 38% were from North America, 21% from Asia, 14% from Africa, 12% from Europe, 9% from South America and 5% from Oceania. By socioeconomic status, 50.4% were from high-income countries (HICs), 22% upper middle-income countries (UMICs), 24.8% low middle-income countries (LMICs), and 2.6% low-income countries (LICs). Most respondents treat both adults and pediatric patients (65%) with an equal number caring for adults or pediatrics only (18% each). The most common drug for managing background pain was paracetamol for all patients and for wound care in pediatrics. Oral and IV opioids were unavailable at 29% and 16% respectively in LMIC/LICs, 36 and 8% in UMICs, and 5.3% for both in HICs. For wound care, IV opiates, paracetamol and benzodiazepines were the most common in HICs and UMICs, while paracetamol was the most common in LMICs/LICs. Ketamine and propofol were used more frequently in UMICs for wound care in adult patients. Non-pharmacological methods were rarely used in adults and in a minority of children.

Most respondents were aware of a pain guideline (72%) and have a pain management protocol at their facility (65%). HICs reported pain at their facility being managed “well” or “very well” (70%) versus half of respondents from UMICs (52%) or LMICs/LICs (50%). Only 11% of respondents from HICs thought pain was managed “somewhat poorly” or “poorly,” versus 16% of UMICs and LMICs/LICs. Those who reported having a protocol for pain management or who were aware of pain guidelines reported better perceived pain control for their patients compared to those who did not (p < 0.05).

**Conclusions:**

Practice around pain control for burn injuries and wound care vary mostly by socioeconomic status and drug availability. Perception of adequate pain control is associated with socioeconomic status, availability of drugs and having a pain protocol.

**Applicability of Research to Practice:**

Burn centers can optimize pain management by establishing standard operating procedures based on existing guidelines. Centers with pain protocols should consider sharing them with other sites to use as a template.

